# Evaluation of an online family history tool for identifying hereditary and familial colorectal cancer

**DOI:** 10.1007/s10689-017-0041-7

**Published:** 2017-09-21

**Authors:** F. G. J. Kallenberg, C. M. Aalfs, F. O. The, C. A. Wientjes, A. C. Depla, M. W. Mundt, P. M. M. Bossuyt, E. Dekker

**Affiliations:** 1 Department of Gastroenterology and Hepatology, Academic Medical Center, University of Amsterdam, Meibergdreef 9, 1105 AZ Amsterdam, The Netherlands; 2 Department of Clinical Genetics, Academic Medical Center, University of Amsterdam, Amsterdam, The Netherlands; 3 Department of Gastroenterology and Hepatology, Onze Lieve Vrouwe Hospital East, Amsterdam, The Netherlands; 4 Department of Gastroenterology and Hepatology, Onze Lieve Vrouwe Hospital West, Amsterdam, The Netherlands; 5 Department of Gastroenterology and Hepatology, Medical Center Slotervaart, Amsterdam, The Netherlands; 6 Department of Gastroenterology and Hepatology, Flevo Hospital, Almere, The Netherlands; 7 Department of Clinical Epidemiology, Biostatistics and Bioinformatics, Academic Medical Center, University of Amsterdam, Amsterdam, The Netherlands

**Keywords:** Family history, Hereditary colorectal cancer, Familial colorectal cancer, Surveillance, Genetic counseling

## Abstract

Identifying a hereditary colorectal cancer (CRC) syndrome or familial CRC (FCC) in a CRC patient may enable the patient and relatives to enroll in surveillance protocols. As these individuals are insufficiently recognized, we evaluated an online family history tool, consisting of a patient-administered family history questionnaire and an automated genetic referral recommendation, to facilitate the identification of patients with hereditary CRC or FCC. Between 2015 and 2016, all newly diagnosed CRC patients in five Dutch outpatient clinics, were included in a trial with a stepped-wedge design, when first visiting the clinic. Each hospital continued standard procedures for identifying patients at risk (control strategy) and then, after a predetermined period, switched to offering the family history tool to included patients (intervention strategy). After considering the tool-based recommendation, the health care provider could decide on and arrange the referral. Primary outcome was the relative number of CRC patients who received screening or surveillance recommendations for themselves or relatives because of hereditary CRC or FCC, provided by genetic counseling. The intervention effect was evaluated using a logit-linear model. With the tool, 46/489 (9.4%) patients received a screening or surveillance recommendation, compared to 35/292 (12.0%) in the control group. In the intention-to-treat-analysis, accounting for time trends and hospital effects, this difference was not statistically significant (p = 0.58). A family history tool does not necessarily assist in increasing the number of CRC patients and relatives enrolled in screening or surveillance recommendations for hereditary CRC or FCC. Other interventions should be considered.

**Electronic supplementary material:**

The online version of this article (doi:10.1007/s10689-017-0041-7) contains supplementary material, which is available to authorized users.

## Introduction

Fifteen to 20% of colorectal cancer (CRC) cases are related to familial or hereditary factors [1–3]. These are mostly familial CRC (FCC), a family-history based diagnosis without a known genetic cause, and to a smaller extent the autosomal dominantly inherited Lynch syndrome, comprising 2 to 4% of CRC cases [1, 4].

Identification of these hereditary and familial factors in CRC patients provides an opportunity to engage in effective screening and surveillance protocols for those patients as well as for their relatives at risk. A detailed assessment of the family history for CRC can help to identify these patients. To judge whether they should be referred for genetic counseling, persons are generally tested against referral criteria, such as the Amsterdam II and the Revised Bethesda criteria [5–7]. However, physicians and nurses might have limited knowledge about these referral criteria and the family history might be incompletely explored [8–15]. Consequently, in daily practice only 10–30% of patients at risk are referred for genetic counseling and for screening and surveillance recommendations [3, 8, 12–17].

To identify persons at an increased risk of FCC or hereditary CRC, we previously developed and validated an online patient-administered questionnaire that provides a full and detailed family history [18]. This questionnaire was incorporated in a family history tool that creates an automated referral recommendation for genetic counseling, in case of suspected Lynch syndrome and for surveillance colonoscopies, in case of FCC [5].

To evaluate its effectiveness, we designed a multicenter trial with a stepped-wedge design, in outpatient clinics for patients with CRC. Each hospital continued standard procedures for identifying patients at risk (control strategy) and then switched to offering the family history tool to included patients (intervention strategy). We anticipated that the implementation of this tool would increase the proportion of newly diagnosed patients with CRC receiving a CRC screening or surveillance recommendation for hereditary CRC or FCC for themselves and/or their relatives, provided by genetic counseling.

## Patients and methods

### Trial design

We conducted a multicenter trial between February 2015 and October 2016, using a stepped-wedge design, in five hospitals in the Netherlands. One is a tertiary academic center, four are general hospitals. Each has a specialized, multidisciplinary outpatient clinic for CRC patients.

A stepped-wedge design is a type of cross-over design in which centers switch protocols in only one direction and at different time points [19]. All hospitals started the control strategy and switched, one by one, to the intervention strategy, in a predetermined order. To allow for a study-duration of 1.5 years, every 9 weeks one hospital switched to the intervention strategy. Before the switch, a training week was initiated, which is not included in the evaluation. All centers kept to the intervention strategy until the last hospital had implemented the family history tool for 9 weeks.

### Control strategy

All hospitals started with their routine practice for identifying patients at risk of hereditary CRC or FCC and referring them to a clinical geneticist. Patients fulfilling criteria for surveillance colonoscopies in case of FCC were also expected to be referred for genetic counseling, after which the genetic counselor provided colonoscopy recommendations. As a reminder, health care providers received an email with the referral criteria for genetic counseling at the start of the control strategy (Table 1). There was no further involvement.

**Table 1:** Referral criteria for a Lynch syndrome suspicion and familial colorectal cancer

Referral criteria for Lynch syndrome	N = 56^g^
1. A patient with colorectal cancer or endometrial cancer < 50 years	10
2. A person^a^ with a first degree relative with colorectal cancer or endometrial cancer < 50 years	5
3. A person^a^ with a family member with a known mismatch repair mutation	0
4. A person^f^ with at least three first or second degree relatives with colorectal cancer or a Lynch syndrome associated tumor^b,c,d^ <70 years	1
5. A patient with colorectal cancer with a synchronous or metachronous colorectal cancer < 70 years	6
6. A patient with colorectal cancer with a synchronous or metachronous Lynch syndrome associated tumor^b^ <70 years	1
7. A patient with colorectal cancer^e^ with a first degree relative with colorectal cancer or a Lynch syndrome associated tumor^b^ <50 years	10
8. A patient with colorectal cancer or a Lynch syndrome associated tumor^b^ with at least two first or second degree relatives with colorectal cancer or a Lynch syndrome associated tumor^b,c,d^, all <70 years	23

Referral criteria for familial colorectal cancer	N = 40^g^
9. A person^f^ with two first degree relatives with colorectal cancer 50–70 years^d^	0
10. A person^f^ with a first degree relative with colorectal cancer 50–70 years and a second degree relative with colorectal cancer <70 years^d^	0
11. A patient with colorectal cancer with a first degree relative with colorectal cancer, both 50–70 years	19
12. A patient with colorectal cancer 50–70 years with a second degree relative with colorectal cancer <70 years	21

Additional referral criteria	N = 1^g^
13. When unclear if genetic testing has been done previously, what the outcome was or whether surveillance recommendations have been given for hereditary or familial colorectal cancer, one can consider to refer the patient to a clinical geneticist	1

^a^With or without colorectal cancer or any other cancer type

^b^Lynch syndrome associated tumors: carcinoma of the endometrium, stomach, small intestines, pancreas, bile ducts, renal pelvis, ureters, ovaries, brain and carcinoma or adenoma of the sebaceous gland

^c^In case one relative has more than one colorectal cancer or Lynch syndrome associated tumor, this counts as two relatives with colorectal cancer or a Lynch syndrome associated tumor

^d^Relatives must all be genetically related (paternal or maternal lineage)

^e^Irrespective of age

^f^A healthy person or a person with colorectal cancer or a Lynch syndrome associated tumor >70 years or a person with any other cancer type irrespective of age

^g^In intervention strategy, 97 criteria were met by 63 patients who had a referral indication based on the family history tool

### Intervention strategy

#### Online family history tool

In the intervention strategy, the family history tool was routinely used, which included an online family history questionnaire and an automated referral recommendation. The development and validation of the questionnaire have been described in detail elsewhere [18]. In short, this is a self-administered online questionnaire for a thorough family history of CRC and Lynch syndrome associated tumors in first- and second-degree relatives (for images of the questionnaire: [18]). It also incorporates the Dutch nationwide referral criteria for genetic counseling in suspected Lynch syndrome or FCC [5]. For the current study we created a tool that, based on the family history and the referral criteria, can automatically recommend on referral. In the tool, we additionally collected data on age, sex, nationality, educational level, native language and reasons for non-participation, if any.

The accuracy of the tool-based referral recommendations was verified by evaluating 250 randomly completed questionnaires as well as 100 questionnaires that were used in the previous validation. A researcher trained in the application of referral criteria assessed if and which criteria applied for each completed questionnaire. His result was compared with the referral recommendation as generated by the family history tool. Whenever discrepancies were detected, the error was corrected. This process was repeated until no errors occurred in all 350 tool-based referral recommendations.

#### Intervention strategy procedures

One researcher presented the study to health care providers in each of the participating outpatient clinics. All nurses who counseled these patients were instructed to routinely invite all eligible patients and to offer the family history tool before or at the time of the first visit. In each hospital, the intervention strategy started with a training week, in which nurses additionally received a manual, a pocket card, and instructions on how and when to use the tool.

Consenting patients received an email with a link to the family history tool and were asked to complete it before or at the first visit, if needed with the help of a nurse. If patients completed the questionnaire at home, the nurse was asked to verify the answers with the patient during the first visit.

Thereafter, the tool-based referral recommendation was verified by the nurse. After considering this recommendation, the nurse or physician responsible for genetic referrals could decide on and arrange the referral. Referral criteria for polyposis syndromes were not evaluated in the tool; hospitals were advised to refer for this indication at their own discretion.

During the intervention strategy, a research fellow (FK) provided background support for inviting and assisting patients to use the tool, verifying answers and reminding nurses to refer patients when indicated.

### Study group

#### Inclusion criteria

CRC patients were eligible if they had a first visit at the outpatient clinic. This included patients with metachronous CRC or local recurrence of a previous CRC. Patients who were seen in more than one participating hospital were only included in the hospital where they received follow-up.

#### Exclusion criteria

It is beneficial to diagnose hereditary CRC or FCC in a patient with CRC before treatment has commenced, as this diagnosis could result in an adjusted treatment (e.g. subtotal colectomy rather than a local resection of a tumor or involving the choice of chemotherapy). Therefore, and also to create a homogeneous study group, we excluded patients who had already received treatment for their CRC or local recurrence before the intake visit.

### Study outcomes

The primary outcome was defined as a recommendation for screening and/or surveillance colonoscopies for patients and/or relatives because of hereditary CRC or FCC, provided by genetic counseling. Data on this outcome parameter were retrieved from genetic counseling letters. Secondary outcomes included types of identified hereditary CRC syndromes or FCC, details about surveillance recommendations, such as who received recommendations (patient and/or relatives) and what specific recommendations were given. We also evaluated the usability of the family history tool for involved nurses and patients by a paper and online evaluation.

### Statistical analysis

We calculated the proportion of participants with a screening and surveillance recommendation for each of the two strategies. As is conventional in the analysis of stepped-wedge study designs, we used a logit-linear model to evaluate the effect of the intervention, taking into account hospital and time-period effects, as additional factors. As the study was performed in five hospitals, we included hospital as a fixed effect. We adhered to the intention-to-treat principle, including all eligible patients in the analysis, but additionally performed an analysis in which patients with a non-CRC lesion were excluded. P-values below 0.05 were assumed to indicate statistically significant effects.

### Sample size calculation

Of all CRC cases, approximately 20% are related to familial or hereditary factors [1–3]. Based on literature, we estimated that with the control strategy 20% of patients at risk would be referred, resulting in a referral rate of 4% of all CRC patients [3, 8, 9, 12, 14, 16, 20]. We anticipated that implementation of the family history tool could increase the latter proportion up to 15%. We assumed we would not identify all patients at risk and anticipated that some patients would not want to be referred [8]. To have 80% power in detecting this difference in a parallel RCT design, a sample size of 111 patients per treatment strategy would be needed. In a stepped-wedge design, to allow for clustering, the total sample size should be multiplied by a design effect, which was calculated at 0.45 (using an estimated intracluster correlation (ICC) of 0.10, as no previously reported ICC for this topic was available) [19]. With these data, a minimum number of 100 patients needed to be recruited.

This study was registered in the Dutch Trial Registry (NTR5398) and on ClinicalTrials.gov (NCT02645084). The study had been approved by the medical ethics committee of the Academic Medical Center in Amsterdam, the Netherlands. This committee decided that the study did not fall under the Medical Research Involving Human Subjects Act in the Netherlands. Per Dutch law, no additional consent was needed for anonymized analyses based on data collected on patients with both strategies. Local ethics committees of all participating hospitals confirmed the local feasibility of this study. All patients who completed the tool in the intervention strategy provided informed consent before participating. This study was carried out in accordance with the Helsinki Declaration [21].

## Results

### Time-frame

**Fig. 1:**
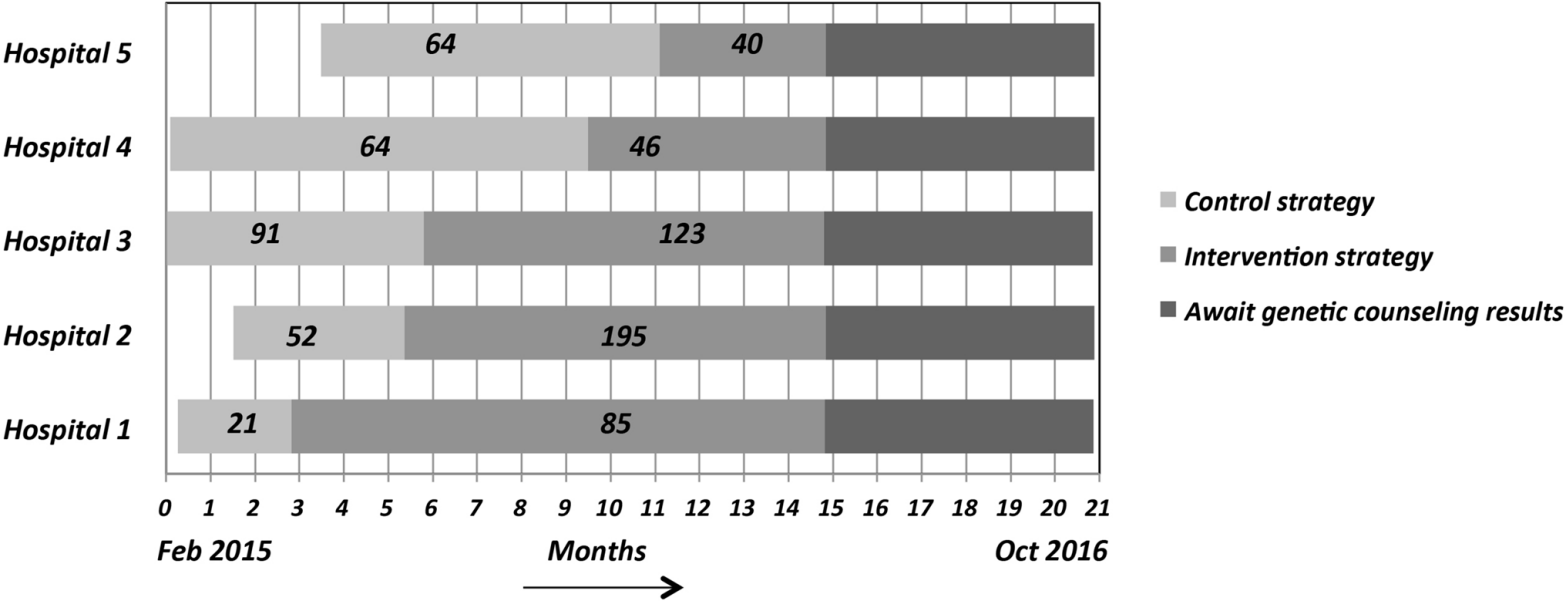
Visualization of the stepped wedge design with numbers of included patients per strategy

Not all hospitals could start the control strategy at the same time, as depicted in Fig. 1. Each hospital switched to the intervention strategy within 3 weeks of the planned date and duration of the control strategy was as projected for each hospital. The fifth hospital was the last to start the control strategy. We decided to extend the duration of the intervention strategy for each hospital until the fifth hospital had finished the full intervention period.

### Patient characteristics

**Fig. 2:**
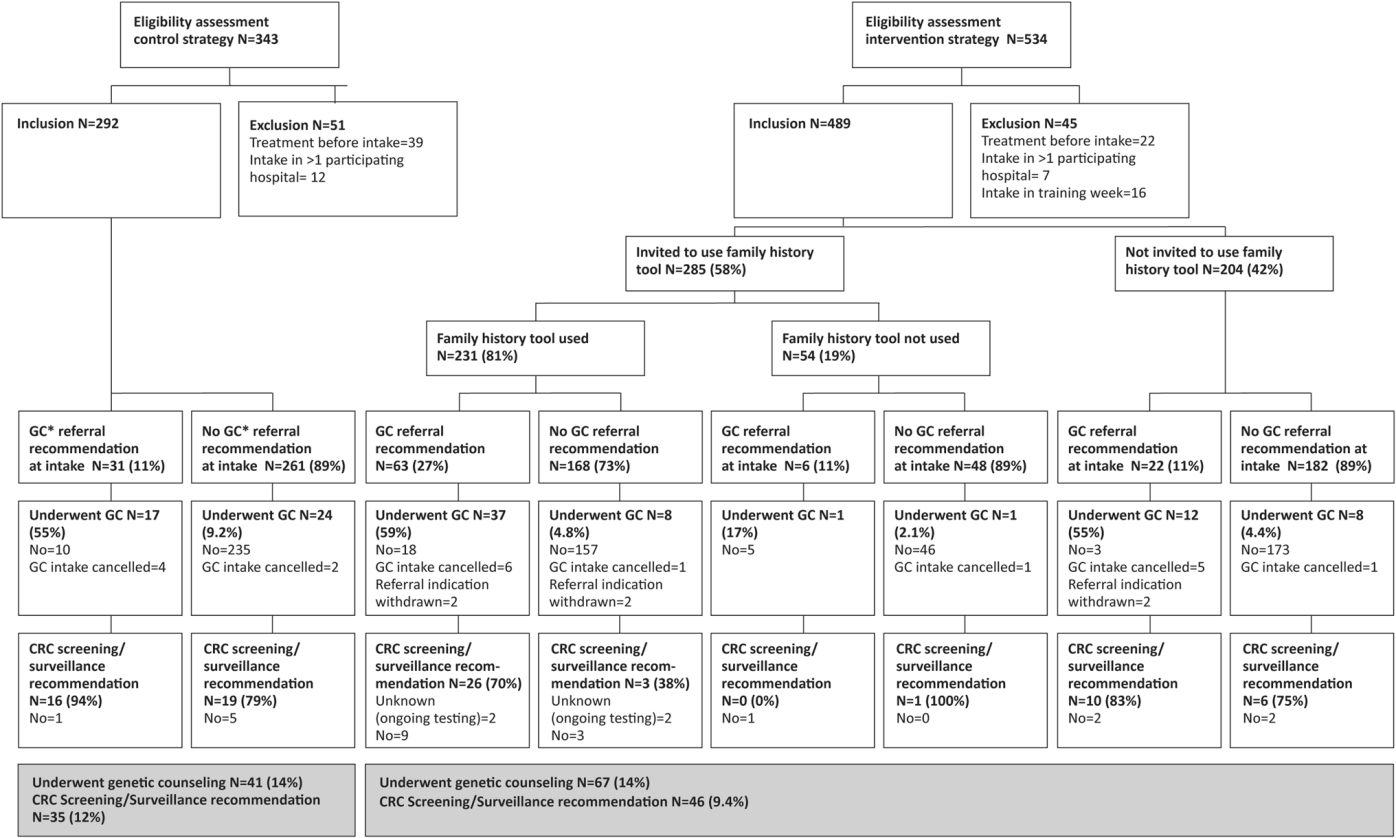
Flowchart illustrating study procedures. *GC genetic counseling

A total of 877 patients were assessed for eligibility during both the control and the intervention periods, of whom 96 could not be included (Fig. 2). The most common reason for exclusion was that patients had already undergone CRC treatment before their first visit to the clinic (n = 61). This resulted in a total number of 781 included patients: 292 in the control strategy and 489 in the intervention strategy. Baseline demographic data between patients in the two strategies were similar, except for the number of patients who had a family history reported in the medical note of the first visit to the outpatient clinic; this number was higher with the intervention strategy (Table 2). After the first visit, histopathology of the presumed CRC lesion turned out benign in 19 patients and showed invasive growth of an ovarian carcinoma in one.

**Table 2:** Baseline characteristics of included patients for the intention-to-treat analysis (n = 781)

Characteristic	Control strategy (n = 292)	Intervention strategy (n = 489)	P value
Mean age (SD, range), years	67 (9.9, 28–92)	67 (9.9, 26–95)	0.55^e^
Male	171 (59%)	265 (54%)	0.23^f^
Pathology after resection			0.64^g^
CRC	284 (97%)	473 (97%)	
No CRC	6^a^ (2.1%)	14^b^ (2.9%)	
No pathology, clinically CRC	2 (0.7%)	2 (0.4%)	
CRC location^c,d^			0.64^g^
Left	182 (64%)	315 (66%)	
Right	103 (36%)	154 (32%)	
Both	1 (0.3%)	6 (1.3%)	
CRC pattern^c^			0.06^g^
First CRC	268 (94%)	450 (95%)	
Synchronous CRC	5 (1.7%)	16 (3.4%)	
Local recurrence	7 (2.4%)	7 (1.5%)	
Metachronous CRC	6 (2.1%)	2 (0.4%)	
Family history reported at intake	179 (61%)	364 (74%)	0.000^f^
FCC/hereditary CRC syndrome at intake			
Yes	7 (2.4%)	9 (1.8%)	0.61^f^
Lynch syndrome	0	0	
Lynch syndrome ruled out	1	3	
Familial colorectal cancer	0	0	
Serrated polyposis syndrome	1	2	
Adenomatous polyposis (FAP, MAP, non-genetic)	2	3	
Syndrome unknown	3	1	
Second opinion/referral	31 (11%)	34 (7.0%)	0.07^f^

^a^Five benign lesions and one patient with an ovarian carcinoma

^b^All benign lesions

^c^Non-CRC lesions not included

^d^Right or left from splenic flexure

^e^2-Sided unpaired t-test

^f^2-Sided chi square test

^g^Fisher’s exact test

### Primary outcome

The percentage of included patients who received a recommendation for screening or surveillance colonoscopies for themselves and/or their relatives, provided by genetic counseling, was 12.0% with the control strategy and 9.4% with the intervention strategy (Table 3). In the logit-linear model analysis, taking into account the hospital and time period effects, the effect of the intervention strategy was not significantly different from zero (conditional odds ratio 0.76; 95% CI 0.29–2.00; p = 0.58). We did not observe significant effects for hospital (p = 0.92) or time-period (p = 0.86). When excluding non-CRC lesions, there was also no significant effect of the intervention (p = 0.64).

**Table 3:** Genetic counseling results

Characteristic	Control strategy (n = 292)	Intervention strategy (n = 489)	P value
Genetic counselor consulted	41 (14%)	67 (14%)	0.89^h^
Diagnosis after genetic testing			
Yes	35 (12%)	46 (9.4%)	0.25^h^
Familial colorectal cancer^a^	26	32	
Lynch syndrome	3	2	
Lynch like syndrome	1	0	
Polyposis syndrome	5^e^	3^f^	
Unknown yet, but definite diagnosis	0	9^g^	
No	6 (2.1%)	17 (3.5%)	
Syndrome ruled out	5	16	
Wrong referral indication	1	1	
Unknown yet^b^	0	4 (0.8%)	
Colonoscopy screening/surveillance recommendation			
Yes	35 (12%)	46 (9.4%)	0.25^h^
For patient	3	3	
For patient and relatives^c^	14	18	
For relatives^c^	18	16	
For patient and possibly relatives^d^	0	9	

^a^Defined as a familial risk of CRC, without a known genetic cause, for which screening or surveillance recommendations are given

^b^Genetic testing ongoing, unknown if a genetic or familial diagnosis will be made

^c^Colonoscopy screening and/or surveillance recommendations for relatives included: Colonoscopy surveillance 1×/5 year from the age of 37, 38, 40 or 45: n = 51, Colonoscopy screening at the age of 45, 50, 55 or 65: n = 14, Colonoscopy surveillance 1×/2 year from the age of 25: n = 1

^d^Genetic testing ongoing that will certainly result in a genetic or familial diagnosis

^e^Including three patients with serrated polyposis syndrome, two with polyposis of unknown origin

^f^Including one patient with polyposis of unknown origin, one with MUTYH associated polyposis and one with serrated polyposis syndrome

^g^Genetic test result unknown yet (either Lynch syndrome or FCC in eight and genetic or non-genetic polyposis in one)

^h^2-Sided chi square test

### Process measures and secondary outcomes

#### Referral for genetic counseling

In the control strategy, a recommendation for referral for genetic counseling was mentioned in the medical report of the first visit for 31 patients (11%) (Fig. 2). Of those, 17 (55%) were indeed seen by a genetic counselor. The remaining 14 patients did not visit a genetic counselor; in most patients (n = 9) the reason was unknown. Of the 261 patients who did not receive a recommendation for referral during the first visit, 24 (9.2%) were referred after the first visit and underwent genetic counseling for various reasons (i.e. a family history of CRC, immunohistochemical abnormalities). In total 41 (14%) patients in the control strategy were seen by a genetic counselor after a median of 3.9 (range 1.2–17.5) months following their first visit to the outpatient clinic.

In the intervention strategy, the family history tool was offered to 285 (58%) patients and was used by 231 (81%) of them. Of those who used the tool, the majority was male (56%), mean age was 66 years, their educational level varied equally between low, moderate or high and 93% had the Dutch nationality. Of these 231 patients, 63 (27%) had a referral indication based on the family history tool (which criteria were met can be found in Table 1) and 37 (59%) of them underwent genetic counseling.

Of the 168 patients who did not have a referral indication based on the family history tool, eight (4.8%) underwent genetic counseling, for various appropriate reasons (i.e. immunohistochemical abnormalities in tumor tissue).

Of the 54 patients who did not use the family history tool after it was offered, 34 provided one or more reasons for non-participation. The most common reasons consisted of not being interested in participation (n = 16) and not feeling like participating in a study now that they were recently diagnosed with cancer (n = 9). Of those 54 patients, two underwent genetic counseling.

In the group of 204 patients who were not offered the tool, 20 (9.8%) patients had undergone genetic counseling. The 67 patients in the intervention strategy who were seen by a genetic counselor (14%) underwent genetic testing after a median of 3.7 months following the CRC intake visit (range 0.5–13.7).

#### Genetic test results

After genetic testing, 35 (12%) of all included patients were diagnosed with a hereditary CRC syndrome or FCC in the control strategy and 46 (9%) with the intervention strategy (p = 0.25) (Table 3). The most common diagnosis in both strategies was FCC (n = 55). With the intervention strategy, a hereditary syndrome or FCC was excluded in more patients after genetic testing (16/67 vs. 5/41). In each strategy, one patient had an inadequate referral for which no further testing was done and in the intervention strategy, four patients were awaiting test results that could result in a genetic diagnosis, but if not, they would not have FCC.

#### Screening and surveillance recommendations

In both strategies, all patients who were diagnosed with hereditary CRC or FCC received a screening and/or surveillance recommendation. Table 3 depicts to whom colonoscopy screening and/or surveillance recommendations were given after genetic counseling (patients, relatives or both). In both the intervention strategy and control strategy, recommendations other than colonoscopy screening and surveillance were also given. Patients received recommendations for relatives to undergo DNA testing for a suspicion of hereditary CRC (n = 12, including the five patients with Lynch syndrome), hereditary breast or ovarian cancer (n = 10), *MUTYH* associated polyposis (n = 1) and hereditary pancreatic cancer syndrome (n = 1). In one family, the suspicion of a hereditary cardiac syndrome was raised, in another family a gastric cancer suspicion for which gastroscopy and H. Pylori screening were recommended and relatives of the patient with Lynch-like syndrome were recommended to undergo H. Pylori testing as well as gynecological surveillance.

#### Evaluation by patients and nurses

Forty-nine patients who had used the family history tool at home provided an evaluation, as well as all eleven involved nurses. These data can be found in Online Resource 1.

## Discussion

As FCC and hereditary CRC syndromes are not always recognized in CRC patients, many persons at risk do not receive appropriate CRC screening and surveillance recommendations. We developed and validated a family history tool that could assist in recognizing CRC patients and their relatives with FCC or a hereditary CRC syndrome. In this multicenter trial with a stepped-wedge design we observed that implementation of this tool in multidisciplinary outpatient clinics for patients with CRC did not increase the proportion of CRC patients with hereditary CRC or FCC receiving surveillance recommendations for themselves or their relatives, compared to standard practice.

What are the potential reasons for the intervention strategy not to be effective in detecting those patients compared to the control strategy? To clarify this, we should first try to explain why the proportion of patients who underwent genetic counseling was similar in both strategies (14%). First, an unexpectedly large proportion of patients received surveillance recommendations in the control strategy (12%, whereas we expected 4%), leaving limited room for improvement. One factor could clarify why the control strategy performed much better than what was previously reported in literature [3, 8, 12–17]. We could have increased awareness by offering a list with the referral criteria to all hospitals at the start of the control strategy.

Secondly, only a disappointing proportion of 58% of eligible patients were offered the family history tool in the intervention strategy. The reasons were not explored, but this could be due to the fact that nurses found the tool time-consuming, study enrolment was not a priority in the first work-up of a patient with CRC, nurses did not always counsel the patient (i.e. after an endoscopically resected T1 carcinoma) and thus not always invited them for participation and selection bias might have occurred (i.e. inviting mainly those with a striking family history of CRC who would be easily recognized without the tool). Moreover, a proportion of patients did not wish to use the tool after it was offered.

A third explanation for the comparable genetic counseling rates may be the false reassurance, resulting from introduction of the family history tool. Physicians may have thought that the tool, that was offered by nurses, would identify everyone with a referral indication. Yet there may also be patients in a ‘grey referral area’, who would usually be referred despite not strictly fulfilling referral criteria, such as a patient with multiple relatives with CRC above the age of 70. Such patients are commonly diagnosed as having a familial risk of CRC. However, in several hospitals nurses were not used to referring patients, as the physician usually did this, and might not have recognized these ‘grey’ referral indications that were not detected by the tool. This is confirmed by the finding that there was a relatively small proportion of patients diagnosed with FCC in the intervention strategy, although this could also have another reason. A year after study commencement (when all hospitals had started the intervention strategy), a new nationwide guideline on the detection of hereditary CRC was introduced, recommending immunohistochemical analysis of mismatch-repair proteins on all CRC cases under the age of 70 years [5]. This could have changed results in the intervention strategy, as patients with a suspect family history but without loss of mismatch-repair proteins might not have been referred, despite still fulfilling criteria for FCC.

Despite the genetic counseling rates being similar, the proportion of referred patients who received screening and surveillance recommendations was (non-significantly) smaller in the intervention strategy. We might clarify this. The family history tool mostly focusses on identifying patients with Lynch syndrome, by taking into account all Lynch syndrome-associated tumors. If genetic testing does not detect Lynch syndrome, especially in patients who fulfill referral criteria based on familial occurrence of extra-colonic Lynch associated tumors, often no surveillance recommendations will be given. This is most likely the reason why in 16/67 (23.9%) referred patients in the intervention strategy a syndrome was excluded, compared to only 6/41 (14.6%) patients in the control strategy. Combining that with the above-mentioned finding that the intervention strategy identified fewer patients with FCC, fewer patients received a surveillance recommendation in the intervention strategy.

Several limitations should be acknowledged. First of all, our aim was to identify more patients and relatives with hereditary CRC syndromes and FCC, although our tool only identified patients with Lynch syndrome and FCC, not those with polyposis syndromes. However, as FCC and Lynch syndrome contribute to the majority of all CRC syndromes and are easily missed due to the lack of specific endoscopic features, we decided that identification strategies should mainly focus on these two groups. For that purpose the tool is accurate, as was previously reported [18]. Another factor that might influence results is that patients could have returned to the referring hospital that was not in our region, resulting in unknown data of genetic counseling. By crosschecking referrals with all genetic centers in the surrounding of the participating hospitals, we believe that having missed referral data within the participating region is unlikely. Besides, this most likely then would have happened equally in both strategies.

Several family history tools that include a referral decision aid have been developed before, but the tool we developed is the first to verify both FCC and all Lynch syndrome associated tumors [22–26]. Only three previous studies have evaluated the effectiveness of a CRC family history tool and compared it to a control setting [22, 25, 26]. An unexpected effect, similar to ours, was seen in a recent Dutch clustered randomized controlled trial in CRC patients [26]. The authors investigated the efficacy of a comparable tool, complemented by a website with information on familial risk of CRC, risk calculators for patients, and a genetic counseling decision support intervention for high-risk patients. This intervention did not improve referral for cancer prevention measures, compared to a control setting. The authors suggested that giving a recommendation for genetic testing through an online application instead of through a physician or nurse could decrease adherence to the recommendation. However, in our study health care providers advised patients on genetic referral, after being informed by the tool. This neither resulted in an effective outcome. In the American Family Healthware Impact Trial, Rubinstein et al. studied the effect of an online tool that stratifies risks for six common diseases, including CRC [25]. In their randomized controlled trial in healthy persons in a primary care setting, the intervention did not result in an increased rate for CRC screening compared to a control setting. Screening rates were already high at baseline, as in our study, resulting in a low power to detect an intervention effect. The third study, the primary care based cluster randomized GRAIDS trial, showed that a family history tool resulted in more patients referred for genetic counseling compared to the current best practice [22]. However, this study involved very small numbers. It thus seems that all those tools, despite a successful validation, do not always work when implemented in real-life health care situations. Reasons for limited success rates should be further evaluated.

Based on our explanations, we can provide suggestions for adjustments in the implementation of the family history tool to improve efficacy. One could consider assessing the proportion of detected hereditary CRC and FCC cases before implementing this tool. If this proportion is less than the generally expected 15–20% of CRC patients with FCC or hereditary CRC, it could be worthwhile to use the tool. To reduce the time and workload needed for implementation, implementation within a pre-existing electronic patient file is desired. To enhance continuity, one or two trained persons can be assigned to apply the tool and decide on genetic referrals, including the above-mentioned ‘grey’ referrals [27, 28]. As we felt that patients as well as health care providers did not prioritize genetic referrals in the early stages of the CRC work-up, these trained persons could implement the tool after surgery instead of at the time of diagnosis and the first visit to the clinic. These adjustments should first be evaluated in comparative effectiveness studies. Moreover, one could consider evaluating its efficacy in other healthcare situations, such as in CRC screening programs or in primary care. In these situations the tool might be more effective, as these persons have not yet been seen by a CRC specialist, resulting in a higher likelihood of detecting unrecognized familial and hereditary factors [29].

We conclude that the family history tool in this multicenter prospective comparative cohort study does not necessarily increase the number of CRC patients and their relatives diagnosed with FCC or hereditary CRC syndromes, and therefore will not automatically increase the number of persons who receive screening or surveillance recommendations to prevent new CRC cases. Other interventions should be considered to facilitate the identification and enrollment of these patients in surveillance programs.

## Electronic supplementary material

Below is the link to the electronic supplementary material.

Supplementary material 1 (DOCX 16 KB)
