# Supplementary material for: Evaluation of an online family history tool for identifying hereditary and familial colorectal cancer
Source: Fam Cancer. 2017 Sep 21;17(3):371–80. doi: 10.1007/s10689-017-0041-7 (PMC5999176; doi:10.1007/s10689-017-0041-7)
Supplement: Supplementary file 1 — Supplementary material 1 (DOCX 16 KB) [file 10689_2017_41_MOESM1_ESM.docx]

**Journal: Familial Cancer**

**Evaluation of an online family history tool for identifying hereditary and familial colorectal cancer**

**F.G.J. Kallenberg, MD; C.M. Aalfs, MD PhD; F.O. The, MD PhD; C.A. Wientjes, MD PhD; A.C. Depla, MD; M.W. Mundt, MD PhD; P.M.M. Bossuyt, PhD; E. Dekker, MD PhD**

**CORRESPONDING AUTHOR**

Prof. dr. E. Dekker; Department of Gastroenterology and Hepatology, Academic Medical Center, University of Amsterdam, Amsterdam, the Netherlands; Address: Meibergdreef 9, 1105 AZ, Amsterdam, the Netherlands; Email: [e.dekker@amc.uva.nl](mailto:e.dekker@amc.uva.nl); Phone: +3120-5664702 ; Fax: +3120-6917033

*Evaluation by patients and nurses*

Forty-nine patients who had completed the family history tool at home provided an evaluation (**Supplementary** **Table 1**). Of these, the majority (n=46; 94%) reported that the tool was easy or very easy to use; 36 (74%) found it pleasant or very pleasant to use the family history tool themselves, in addition to family history taking by the health care provider. Fourteen patients (29%) had contacted relatives to clarify unknown family history data. In an additional comment, one patient mentioned that the tool was confronting, now that she was diagnosed with cancer. Three comments were made concerning relatives; one patient said that contacting relatives for missing data was not possible as most were deceased, one patient did not manage to do so and one did not want to contact relatives.

All eleven involved nurses completed an evaluation form (**Supplementary** **Table 2**). Six of them said that the physician collected this data before the start of the study, one nurse did not know who collected family history, and four nurses reported that they collected family history before the start of this study. Of these four, all reported that the tool gave them more confidence in the detection of hereditary CRC and FCC. All four also mentioned that the use of this tool required more time and work. In an additional comment one nurse reported that verifying completed questionnaires with the patients was the most time consuming. Nine nurses said the use of the tool was easy (8) or very easy (1) and all eleven nurses said they thought it would be a good idea to implement the family history tool in their daily practice. Furthermore, family history collection was definitely or probably done better according to nine and eight nurses said that the tool led to a better detection of hereditary CRC and FCC compared to before.

**Supplementary Table 1. Evaluation of the family history tool by 49 patients who used it at home**

| **Question** | **N (%)** |
| --- | --- |
| **Use of the tool**  Easy or very easy  No opinion  Difficult or very difficult | 46 (94)  1 (2)  2 (4) |
| **Using tool at home in addition to physician’s family history collection**  Pleasant or very pleasant  No opinion  Unpleasant or very unpleasant | 36 (74)  11 (22)  2 (4) |
| **Approach relatives for missing data**  No missing data  Yes  No | 15 (30)  14 (29)  20 (41) |

**Supplementary Table 2. Evaluation of the family history tool by all 11 involved nurses**

| **Question** | **N (%)** |
| --- | --- |
| **Family history collection done by nurse before start of the study**  Yes  No, it was done by the physician  No, but unknown who did it | 4 (36)  6 (55)  1 (9) |
| **Use of the family history tool**  Easy or very easy  No opinion  Difficult or very difficult | 10 (91)  1 (9)  0 |
| **Recommend family history tool in daily practice**  Likely or definitely  No opinion  Unlikely or definitely not | 11 (100)  0  0 |
| **Family history collection better with family history tool**  Likely or definitely  No opinion  Unlikely or definitely not | 9 (82)  1 (9)  1 (9) |
| **Hereditary CRC/FCC detection better with family history tool**  Likely or definitely  No opinion  Unlikely or definitely not | 8 (73)  3 (27)  0 |
| **Confidence about detecting/excluding hereditary CRC/FCC with family history tool ^a^**  More than before  As much as before  Less than before | 4 (100)  0  0 |
| **Amount of time/work spent on family history and referral since introduction of family history tool ^a^**  More or much more  As much as before  Less or much less | 4 (100)  0  0 |

^a^ Only applicable for the four nurses who collected family history before the start of the study
